# Performance of eHealth Data Sources in Local Influenza Surveillance: A 5-Year Open Cohort Study

**DOI:** 10.2196/jmir.3099

**Published:** 2014-04-28

**Authors:** Toomas Timpka, Armin Spreco, Örjan Dahlström, Olle Eriksson, Elin Gursky, Joakim Ekberg, Eva Blomqvist, Magnus Strömgren, David Karlsson, Henrik Eriksson, James Nyce, Jorma Hinkula, Einar Holm

**Affiliations:** ^1^Department of Medical and Health SciencesLinköping UniversityLinköpingSweden; ^2^Department of Behavioural Sciences and LearningLinköping UniversityLinköpingSweden; ^3^Department of Computer and Information ScienceLinköping UniversityLinköpingSweden; ^4^National Strategies Support DirectorateANSER/Analytic Services Inc.Arlington, VAUnited States; ^5^Department of Geography and Economic HistoryUmeå UniversityUmeåSweden; ^6^Department of AnthropologyBall State UniversityMuncie, INUnited States; ^7^Department of Clinical and Experimental MedicineLinköping UniversityLinköpingSweden

**Keywords:** influenza, infectious disease surveillance, Internet, eHealth, Google Flu Trends, telenursing call centers, website usage, open cohort design, public health

## Abstract

**Background:**

There is abundant global interest in using syndromic data from population-wide health information systems—referred to as eHealth resources—to improve infectious disease surveillance. Recently, the necessity for these systems to achieve two potentially conflicting requirements has been emphasized. First, they must be evidence-based; second, they must be adjusted for the diversity of populations, lifestyles, and environments.

**Objective:**

The primary objective was to examine correlations between data from Google Flu Trends (GFT), computer-supported telenursing centers, health service websites, and influenza case rates during seasonal and pandemic influenza outbreaks. The secondary objective was to investigate associations between eHealth data, media coverage, and the interaction between circulating influenza strain(s) and the age-related population immunity.

**Methods:**

An open cohort design was used for a five-year study in a Swedish county (population 427,000). Syndromic eHealth data were collected from GFT, telenursing call centers, and local health service website visits at page level. Data on mass media coverage of influenza was collected from the major regional newspaper. The performance of eHealth data in surveillance was measured by correlation effect size and time lag to clinically diagnosed influenza cases.

**Results:**

Local media coverage data and influenza case rates showed correlations with large effect sizes only for the influenza A (A) pH1N1 outbreak in 2009 (*r*=.74, 95% CI .42-.90; *P*<.001) and the severe seasonal A H3N2 outbreak in 2011-2012 (*r*=.79, 95% CI .42-.93; *P*=.001), with media coverage preceding case rates with one week. Correlations between GFT and influenza case data showed large effect sizes for all outbreaks, the largest being the seasonal A H3N2 outbreak in 2008-2009 (*r*=.96, 95% CI .88-.99; *P*<.001). The preceding time lag decreased from two weeks during the first outbreaks to one week from the 2009 A pH1N1 pandemic. Telenursing data and influenza case data showed correlations with large effect sizes for all outbreaks after the seasonal B and A H1 outbreak in 2007-2008, with a time lag decreasing from two weeks for the seasonal A H3N2 outbreak in 2008-2009 (*r*=.95, 95% CI .82-.98; *P*<.001) to none for the A p H1N1 outbreak in 2009 (*r*=.84, 95% CI .62-.94; *P*<.001). Large effect sizes were also observed between website visits and influenza case data.

**Conclusions:**

Correlations between the eHealth data and influenza case rates in a Swedish county showed large effect sizes throughout a five-year period, while the time lag between signals in eHealth data and influenza rates changed. Further research is needed on analytic methods for adjusting eHealth surveillance systems to shifts in media coverage and to variations in age-group related immunity between virus strains. The results can be used to inform the development of alert-generating eHealth surveillance systems that can be subject for prospective evaluations in routine public health practice.

## Introduction

There has been abundant global interest in the use of interactive health information technology—referred to as eHealth systems—to improve the effectiveness of infectious disease surveillance [1]. However, similar to other eHealth applications, surveillance systems based on eHealth data must live up to two potentially conflicting requirements: they must be both evidence-based and adapted to how people live within their specific environments [2]. In other words, eHealth systems’ development, specification, and evaluation in any setting are dependent on the infrastructure, habits, and culture in that setting and at that point in time. This fact must be taken in regard when transferring an eHealth system for use at other locations and periods of time. An example of eHealth technology employed in infectious disease surveillance is Google Flu Trends (GFT), an Internet-based software system that uses aggregated data from the Google search engine to estimate influenza activity [3]. Early studies comparing GFT data to census region influenza-like illness (ILI) data in the United States [4] and prospectively collected sentinel data from two systems in Australia demonstrated strong correlations [5]. However, similar evaluations performed during the influenza A (A) pH1N1 circulation in 2009 in New Zealand [6], Singapore [7], and the United States [8] reported inconsistencies between the GFT and ILI data. One hypothetical reason for such inconsistencies is that mass media coverage of an influenza outbreak can influence behavior by motivating the layperson to seek additional information [9]. Another possible reason may be that because the proportion of adolescents and young adult cases varies between influenza seasons [10], age-related information technology and Internet use consequently impacts eHealth surveillance performance [11].

The purpose of this study is to support evidence-based strategies for eHealth system development in infectious disease control. The primary study objective is to examine correlations between GFT data, telenursing call data, health service webpage usage data, and influenza case rates during seasonal and pandemic influenza outbreaks. The secondary objectives are to investigate associations between eHealth data, the media coverage of influenza outbreaks, and the interaction between the circulating influenza strain(s) and age-related variations in population immunity. To avoid climate and sociogeographic factors affecting the analyses [12,13], all data were collected from one Swedish county (Östergötland, population 427,000) located in South-East Sweden. The entire county population is covered by an electronic health data repository maintained by the county council to systematically and continuously insure the quality of service [14]. The repository collects data from all health care encounters provided in the county at primary and secondary levels, as well as eHealth data from calls made by the county residents to the nation-wide telenursing service, GFT outputs relevant for the county, and data from visits at the county council website (Table 1).

**Table 1:** eHealth systems in Östergötland county, Sweden, investigated in the study.

eHealth systems	Description
Google Flu Trends (GFT)	The GFT service was launched by the Web search engine provider Google in 2008 to track changes in the volume of online search queries related to influenza or its symptoms [3]. For Sweden, the GFT data on Web queries are derived at country and regional levels from a pool of search terms that relate to symptoms, remedies, and complications of influenza and generate a trend that closely correlates data on ILI.
Swedish “Healthcare Direct/1177” telenursing service	Telenursing is defined as computer-supported call centers staffed by registered nurses who perform counselling and patient triage as a means of augmenting self-care support and regulating patient access to medical services [15]. The Swedish national telenursing system “Healthcare Direct” is a 24/7, nurse-led, telephone advice service with one country-wide phone number (1177). Specially-trained nurses use a computerized decision-aid program and an Electronic Health Record (EHR) system for every call. After each call, a chief complaint from a fixed-field terminology register is recorded in the EHR.
Swedish “Healthcare Direct/1177” Internet health information service	“Healthcare Direct/1177” also maintains a national Internet-based health information service, with a specific website for each participating county council. This service consists of general information pages, arranged according to topics such as symptom evaluation guidelines and disease facts and self-management information. Each website is also connected to a Web traffic analysis facility, which at the time of the study was Google Analytics (GA).

## Methods

### Study Design

The study used an “open cohort” design based on the total population of Östergötland county. Open cohort denotes that new cohort members were included by birth or moving into the county and other members were excluded when passing away or moving out from the county as the cohort follow-up progressed. To update the open study cohort, annual aggregated data on the sex, age, and residence of the county population were collected each year from Statistics Sweden. In accordance with Swedish legislation (SFS 2008:355), personal identifiers were removed from the records. The start and end time of an influenza outbreak was defined as 8 incident ILI cases diagnosed in the county during a floating seven-day period. The study design was approved by the Regional Research Ethics Board in Linköping (dnr. 2012/104-31).

### Data Collection

Data on clinical influenza cases and eHealth data were collected between November 2007 and April 2012 using the electronic health data repository maintained by the county council. Data from the clinical laboratories were, for this study, collected during the period 2009-01-01 to 2010-09-15. Influenza cases were identified by the ICD-10 codes for influenza (J10.0, J10.1, J10.8, J11.0, J11.1, J11.8). Influenza-related telenursing calls were identified by the chief complaint codes associated with influenza symptoms: dyspnea, fever (child, adult), cough (child, adult), sore throat, lethargy, syncope, dizziness, and headache (child, adult), from the fixed-field terminology register. GFT data for the study period were collected using a Google account to download data on Google searches from Östergötland county on seasonal and pandemic (for the 2009-2010 outbreak) influenza to a database. The downloaded dataset did not consist of absolute search rate data, but consisted of influenza Web search data normalized with regard to total Web search volumes by the GFT software. Usage data from the county council webpages were collected beginning in May 2009. For technical reasons associated with a change of software providers, data could not be retrieved for the 2010-2011 influenza season. Usage data from January 2012 for the Web-based information service, measured by the numbers of visits of a certain type of page, were collected by directly accessing the Google Analytics (GA) Web traffic analysis instances and by retrieving data through its application programing interface. The Web traffic data contain information about the location of the Web user based on the IP address of the user’s computer (at the granularity of counties). Filters can also be applied based on keywords in page titles or page addresses (URLs) or by pre-selecting certain URLs. Page type refers to the kind of content the page contains, such as factual information about influenza, self-care information, frequently asked questions and answers, or news pages, respectively. Data on media coverage of influenza outbreaks were collected from the online database of the largest newspaper in the county (Östgöta Correspondenten). The database was searched for articles with the term “influenza” (influensa) for the period between November 2007 and April 2012.

### Data Analysis

The influenza case data defined by clinical diagnoses were validated against case data from the microbiological laboratories for the period 2009-01-01 to 2010-09-15. In these analyses, both datasets were separately adjusted for week-day effects on care resource utilization. The correlations between the number of cases reported each day from the clinical and laboratory data were analyzed with a 0–6 day lag. Thereafter, to analyze the relative distribution of influenza cases between age groups, the Relative Illness Ratio (RIR), which is the ratio of the percentage of individuals with an influenza diagnosis in a given age group to the percentage of the general population belonging to the same age group, was computed for each age group and outbreak (circulating virus type) using the formula:

RIR_i_=(C_i_ / C) / (N_i_ / N)

where C_i_is the number of influenza cases in age group i, C is the number of influenza cases in total, N_i_is the population in age group i, and N is the total population in Östergötland county. Further, 95% confidence intervals were calculated for each RIR, using a method based on normal approximation of the Poisson distribution.

In the main analyses of associations between eHealth and influenza case data, Pearson’s correlation coefficients (*r*) were examined to compare influenza case rates with the eHealth data sources (ie, GFT data and all possible combinations of telenursing chief complaints and website page visits with a 2-week time lag to influenza case rates). The three groupings of chief complaints and combinations of website page types, respectively, with the strongest correlation to the influenza case rate for each time lag were listed. The chief complaint grouping and website page combination with the largest correlation effect size were chosen to be used in the final analyses. Separate analyses were performed of correlations between media reports (weekly rate of articles in the regional newspaper mentioning influenza), influenza case rates, and the eHealth data sources (GFT data and all possible combinations of telenursing chief complaints and website page visits with a 2-week time lag to media reports) respectively. The level of statistical significance was set to *P*<.05. To denote the strength of correlations, limit values were applied as suggested by the Cohen Scale [16]. This scale defines small, medium, and large effect sizes as .10, .30, and .50 respectively. The analyses were performed using SPSS version 19, R Statistical Software version 2.15.2, and Minitab Statistical Software version 16.1.1.

## Results

### Overview

The results from the validation analyses showed correlations with large effect sizes between the number of clinically diagnosed influenza cases per day and the corresponding number of cases verified daily by microbiological analyses during the validation period. The correlation with largest effect size (*r*=.63, *P*<.001) was observed between the clinically and the microbiologically verified cases with a two-day lag.

The five-year study period covered four winter influenza seasons and one pandemic outbreak; winter influenza seasons occurred between 2008-01-21 to 2008-04-30 (B and A H1) and 2008-12-24 to 2009-03-30 (A H3N2), the pandemic outbreak lasted from 2009-08-21 to 2009-12-22 (A pH1N1), and the two winter influenza seasons occurring after the pandemic lasted from 2010-12-21 to 2011-04-21 (B and A pH1N1) and 2012-01-09 to 2012-04-14 (A H3N2).

**Figure 1:**
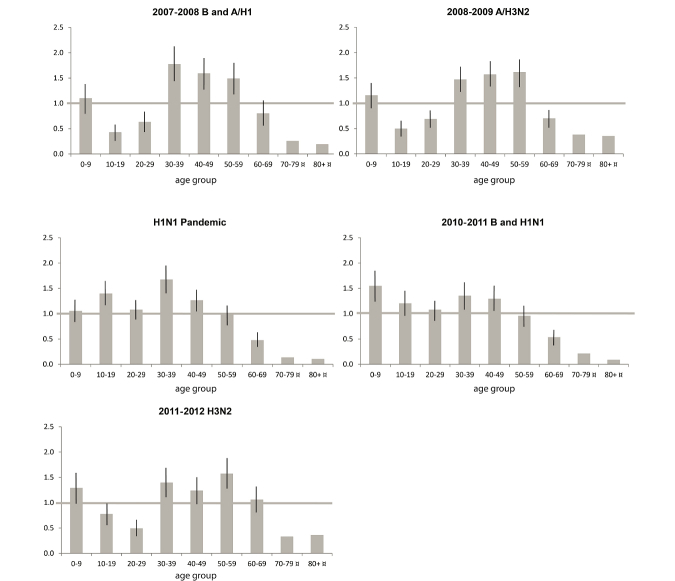
Relative infection ratios (RIRs) with 95% confidence intervals for influenza outbreaks between 2007 and 2012 in Östergötland county displayed by decennial age groups. ¤ Too few observations to allow statistical analysis.

The relative infection ratios for the different age groups and outbreaks are displayed in Figure 1. Higher-than-expected proportions of cases were distributed in the middle-aged groups (30-39 and 40-49 years) during all outbreaks, while lower-than-expected proportions of cases among adolescents and young adults (10-19 and 20-29 years) were recorded for those winter influenza seasons when the pandemic A pH1N1 virus was not circulating.

### Correlations Between Local Media Coverage, Influenza Rates, and eHealth Data

**Figure 2:**
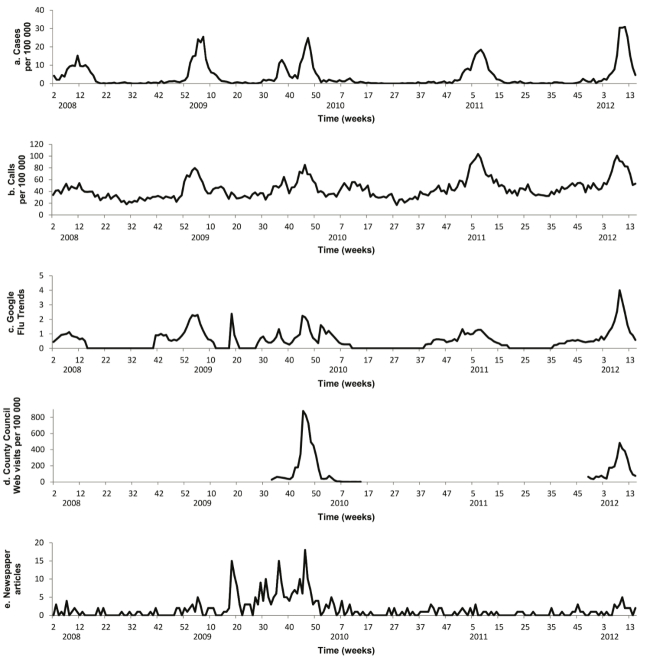
Display of (a) daily rates of influenza cases, (b) daily rates of telenursing calls for indicator chief complaints (fever and syncope), (c) Google Flu Trends output, (d) Influenza-specific website usage at local health service provider, and (e) articles mentioning influenza in major regional newspaper. All data were collected from Östergötland County, Sweden, from November 2007 to April 2012.

The correlations between local media coverage data and influenza case rates showed large effect sizes only for the A pH1N1 outbreak in 2009 (*r*=.74, 95% CI .42-.90; *P*<.001), and the severe seasonal A H3N2 outbreak in 2011-2012 (*r*=.79, 95% CI .42-.93; *P*=.001). For both outbreaks, media coverage preceded case rates by one week. In addition, media reports about influenza showed a peak for weeks 18-22 of 2009 that coincided with a sharp increase in GFT activity, but these peaks had no correspondence with influenza rates or telenursing data (Figure 2). Correlations between media coverage and GFT showed large effect sizes for the seasonal outbreak in 2008-2009 (*r*=.62, 95% CI .15-.86; *P*=.014), the A pH1N1 outbreak in 2009 (*r*=.69, 95% CI .35-.87; *P*=.001), and the seasonal A H3N2 outbreak in 2011-2012 (*r*=.77, 95% CI .39-.93; *P*=.002). The strongest correlations were found for no time lag except for the seasonal outbreak in 2012, when GFT activity preceded media coverage by one week. Neither telenursing data nor the data from health service provider webpages showed statistically significant correlations with the local media coverage data.

### Correlations Between GFT and Influenza Case Data

The correlations between GFT and influenza case data showed large effect sizes for all outbreaks, varying between *r*=.69 (95% CI .22-.90), *P*=.010, for the B and A H1 outbreak in 2007-2008 to *r*=.96 (95% CI .88-.99), *P*<.001, for the seasonal A H3N2 outbreak in 2008-2009 (Table 2). The time lag between GFT and influenza case data decreased from two weeks during the first outbreaks to one week from the 2009 A pH1N1 pandemic, with GFT data preceding influenza case data.

**Table 2:** Associations on a weekly basis between GFT data and influenza case data displayed by the correlation coefficient *r* (95% CI), for the five influenza outbreaks observed in Östergötland county, Sweden, during the study period 2007-2012.

Outbreak time lag	2007-2008	2008-2009	2009	2010-2011	2011-2012
	B and A H1	A H3N2	A pH1N1	B and A pH1N1	A H3N2
(weeks)	(15 weeks)	(15 weeks)	(19 weeks)	(18 weeks)	(14 weeks)
	*r* (95% CI)	*r* (95% CI)	*r* (95% CI)	*r* (95% CI)	*r* (95% CI)
0	ns^c^	.66 (.23-.88) *P*=.007	.79 (.53-.92) *P*<.001	.57 (.14-.82) *P*=.013	.83 (.54-.95) *P*<.001
1^a^	ns	.86 (.61-.96) *P*<.001	.92 (.79-.97) *P*<.001	.75 (.42-.90) *P*=.001	.95 (.83-.98) *P*<.001
2^b^	.69 (.22-.90) *P*=.010	.96 (.88-.99) *P*<.001	.69 (.31-.88) *P*=.002	.81 (.53-.93) *P*<.001	.83 (.50-.95) *P*=.001

^a^Time lag 1 week=Influenza diagnoses 1-week time shift, ie, people first Google the terms “influenza” or “swine flu” and 1 week later visit the health services.

^b^Time lag 2 weeks=Influenza diagnoses 2-week time shift, ie, people first Google the terms “influenza” or “swine flu” and 2 weeks later visit the health services.

^c^ns=not statistically significant

### Correlations Between Telenursing Call Data and Influenza Case Data

The correlations between telenursing data and influenza case data showed large effect sizes for all outbreaks except for the seasonal B and A H1 outbreak in 2007-2008. The preceding time lag for the optimal correlation changed from two weeks for the seasonal A H3N2 outbreak in 2008-2009 to none for the A p H1N1 outbreak in 2009 and one week for the most recent two seasonal outbreaks (Table 3). The telenursing chief complaints included in combinations showing the correlations with largest effect size to influenza case data during most seasons were fever (child, adult) and syncope.

**Table 3:** Associations on a weekly basis between telenursing call data and influenza case data displayed by the correlation coefficient *r* (95% CI), for the five influenza outbreaks observed in Östergötland county, Sweden, during the study period 2007-2012.

Outbreak time lag	2007-2008	2008-2009	2009	2010-2011	2011-2012
	B and A H1	A H3N2	A pH1N1	B and A pH1N1	A H3N2
(weeks)	(15 weeks)	(15 weeks)	(19 weeks)	(18 weeks)	(14 weeks)
	*r* (95% CI)	*r* (95% CI)	*r* (95% CI)	*r* (95% CI)	*r* (95% CI)
0	ns^c^	ns	.84 (.62-.94) *P*<.001	.91 (.77-.97) *P*=.001	.90 (.70-.97) *P*=.008
1^a^	ns	.81 (.48-.94) *P*=.001	.80 (.52-.92) *P*<.001	.95 (.86-.98) *P*<.001	.97 (.91-.99) *P*<.001
2^b^	ns	.95 (.82-.98) *P*<.001	ns	.88 (.69-.96) *P*=.001	.93 (.77-.98) *P*=.002

^a^Time lag 1 week=Influenza diagnoses 1-week time shift, ie, people first call Healthcare Direct/1177 and 1 week later visit the health services.

^b^Time lag 2 weeks=Influenza diagnoses 2-week time shift, ie, people first call Healthcare Direct/1177 and 2 weeks later visit the health services.

^c^ns=not statistically significant

### Correlations Between Log Data From County Council Website and Influenza Case Data

During the 2009 A pH1N1 pandemic, the correlation with the largest effect size between log data from the county council website and influenza case data (*r*=.75, 95% CI .45-.90; *P*=.004) was noted for the website section “Influenza self-care and treatment” with no time lag, while the correlation with one week preceding lag (website visit followed by health care visit) for data from the section “Influenza facts” was only slightly smaller (*r*=.74, 95% CI .42-.90; *P*=.006). For the seasonal A H3N2 outbreak in 2012, the correlation with the largest effect sizes were noted for log data from the website section “Influenza self-care and treatment” with no time lag to influenza cases (*r*=.94, 95% CI .84-.98; *P*<.001) and “Influenza facts” with one week preceding lag (*r*=.94, 95% CI .84-.98; *P*<.001).

### Correlations Between GFT Data, Telenursing Data, and Log Data From County Council Website

The correlations between GFT data and telenursing data showed large effect sizes for all outbreaks (Table 4). During the seasonal outbreaks in 2007-2008 and 2008-2009, the telenursing data preceded the GFT data by one week, while during the A pH1N1 in 2009 and the following seasonal outbreaks the GFT data either preceded the telenursing data by one week or corresponded in time. The correlations between GFT data and log data from the county council website showed large effect sizes for the two outbreaks for which data were available. For both the A pH1N1 outbreak in 2009 (*r*=.87, 95% CI .69-.95; *P*<.001) and the seasonal A H3N2 outbreak in 2011-2012 (*r*=.96, 95% CI .90-.99; *P*<.001), the data sources showed optimal correlation when no time lag was introduced. Also, the correlations between telenursing data and log data from county council website showed large effect sizes for the outbreaks for which data were available. For the 2009 A pH1N1 outbreak, the data sources showed optimal correlation (*r*=.88, 95% CI .71-.95; *P*<.001) when no time lag was introduced, while for the seasonal A H3N2 outbreak in 2011-2012 the optimal correlation (*r*=.95, 95% CI .84-.99; *P*<.001) was observed when telenursing data was relocated to precede the log data from county council website with one week lag.

**Table 4:** Associations on a weekly basis between GFT data and telenursing call data displayed by the correlation coefficient *r* (95% CI), for the five influenza outbreaks observed in Östergötland county, Sweden, during the study period 2007-2012.

Outbreak time lag	2007-2008	2008-2009	2009	2010-2011	2011-2012
	B and A H1	A H3N2	A pH1N1	B and A pH1N1	A H3N2
(weeks)	(15 weeks)	(15 weeks)	(19 weeks)	(18 weeks)	(14 weeks)
	*r* (95% CI)	*r* (95% CI)	*r* (95% CI)	*r* (95% CI)	*r* (95% CI)
−1^a^	.88 (0.65-0.96) *P*=.012	.92 (0.77-0.98) *P*=.001	ns	ns	.90 (0.69-0.97) *P*=.011
0	ns^d^	.88 (0.68-0.96) *P*=.008	.77 (0.49-0.91) *P*=.034	.85 (0.63-0.94) *P*=.005	.94 (0.83-0.98) *P*=.001
1^b^	ns	ns	.87 (0.68-0.95) *P*=.001	.94 (0.83-0.98) *P*<.001	.87 (0.60-0.96) *P*=.032
2^c^	ns	ns	ns	.81 (0.53-0.93) *P*=.016	.86 (0.56-0.96) *P*=.040

^a^Time −1 week=Healthcare Direct/1177 1-week time shift, ie, people first call Healthcare Direct/1177 and then use GFT one week later.

^b^Time lag 1 week=telenursing data 1-week time shift, ie, people first use GFT and then call Healthcare Direct/1177 one week later.

^c^Time lag 2 weeks=telenursing data 2-week time shift, ie, people first use GFT and then call Healthcare Direct/1177 two weeks later.

^d^ns=not statistically significant

## Discussion

### Principal Findings

The primary objective of this study was to examine the performance of data from GFT, telenursing call centers, and health service provider websites in influenza surveillance, while a secondary objective was to investigate associations between eHealth data, media coverage, and the interaction between circulating influenza strain(s) and age-related population immunity. We found correlations with large effect sizes between data from these eHealth sources and influenza case rates for both seasonal and pandemic outbreaks, with the exception of telenursing data during the seasonal B and A H1 outbreak in 2007-2008. A utilization study of the Swedish telenursing service reported that young adults living independently constituted a large group of callers [17]. A contributing explanation for the inferior performance of the telenursing data in 2007-2008 can therefore be that this winter influenza season comprised only a small proportion of young adults (Figure 1), which may have led to comparatively fewer individuals with influenza symptoms contacting the telenursing service. Regarding GFT, the findings are consistent with previous studies conducted at the national and state levels that have reported correlations with large effect sizes between GFT and ILI case data [18-20] and a recent study that reported large effect size correlations with ILI case rates at the local level [21]. Similar to the telenursing data, GFT showed in this study lower correlations with influenza case rates for the 2007-2008 and 2008-2009 winter seasons when comparatively fewer young adults were diagnosed with influenza. Interestingly, we also found that website usage data specified at page level (as compared to search query data) from the local health service provider showed a performance similar to GFT for the two influenza seasons for which these data were collected. Importantly, the webpages showing the best performance contained information about self-care and influenza facts, rather than general outbreak updates or materials concerning vaccination.

Although the correlations between the eHealth data and influenza case rates showed large effect sizes throughout the study period, the time lag between signals in eHealth data and increases in case rates changed. Our results thereby indicate important distinctions in the performance of eHealth systems for influenza surveillance. While the eHealth data tended to precede influenza rates by two weeks during the first two seasonal outbreaks, the time lag was reduced to one week or none from the pandemic outbreak in 2009 onwards. These findings correspond with previous studies [6,7], which concluded that eHealth data associated with infections emerge not only from personal need, but also from an associated general interest. In other words, a layperson’s interest in influenza epidemiology may be triggered by media publicity. Such differentiation is not only relevant for pandemic outbreaks. We found in this study that the local media coverage data preceded influenza case data by one week during the pandemic outbreak in 2009 and the severe winter influenza season in 2011-2012. One interpretation of these observations is that the media coverage reflected a speculative “early warning” viewpoint on the outbreak rather than reports of case rates. Nonetheless, when an early warning is ambiguous or poorly validated, the public may form misperceptions of risks that, consequently, misdirect their behavior [22]. Among the studied eHealth systems, bias from media coverage seemed to influence GFT in particular, as this was the only source that showed large effect size correlations with media coverage data. This interpretation is further supported by the fact that the local media coverage data and the GFT data displayed peaks without corresponding increases in influenza case rates in April-May 2009, a period when the “swine flu” outbreak was highlighted in international media. Additionally, during the seasonal outbreaks in 2007-2008 and 2008-2009, the telenursing data preceded the GFT data by one week, while during the A pH1N1 outbreak in 2009 and the following winter influenza seasons the GFT data either preceded the telenursing data by one week or corresponded in time. Deviances in GFT activity during seasonal outbreaks associated with shifts in media coverage have also been reported from the United States in 2012-2013 influenza season [23], when the GFT estimate for the national peak was almost double that of the CDC. This deviance was attributed to widespread media coverage. Evidently, eHealth data originating from self-care or family care needs have the highest validity when used in infectious disease surveillance. According to agenda-setting theory, mass media have an important influence on what issues the public consider to be important [24]. If a differentiation cannot be made between eHealth data driven by a widespread “lay” epidemiological interest and personal need or care of related individuals, the corresponding data sources risk losing their value in supplementing traditional infectious disease surveillance. Thus, ensuring that Internet data reflect true influenza incidence requires cross-validation with infection-specific data. One strategy is to use telenursing data for real-time validation of Internet data sources, since data on complaints such as fever and cough from telenursing services are less likely to be affected by media publicity than data reflecting Internet activity. However, telenursing data may still not be sufficient for cross-validation if the circulating virus strains mainly affect the older age groups, since the telenursing service utilization in these groups is lower. An alternative is to use data on over-the-counter (OTC) drug sales [25], but the availability of these data in real time may be limited in many countries. Additionally, eHealth data sources can be analyzed in novel ways, such as using multivariate time series methods [26], to obtain improved situational awareness and predictive performance. Nonetheless, these observations suggest that regular validation of the syndromic data sources against clinical and laboratory data is necessary when using eHealth data in influenza surveillance.

### Strengths and Limitations

The strength of this study is that it compares three eHealth data streams over a five-year period, including both winter influenza seasons and a pandemic influenza outbreak, and identifies cross-correlations and time lags for the different outbreaks. The Östergötland population is fairly representative for Sweden as a whole, making it possible, although with care, to generalize the results to communities in settings with similar North-European population and geographical characteristics. Although 15% of the Swedish population is foreign-born, the immigrants have arrived mainly from European countries and are well integrated in the Swedish community. There are no reasons to assume that these immigrants’ utilization of health care or eHealth resources differ from the remaining population to an extent that would affect the use of eHealth data for influenza surveillance. Moreover, there are small differences in health care utilization between urban and rural areas in Sweden [10] and the eHealth resources evaluated in this study are evenly accessible in all Swedish counties. However, the study also has important limitations that should be considered when interpreting the results. First, influenza cases were defined by clinical diagnosis, and microbiological validation was restricted to a limited period of the study. However, the effect size of the correlation between the microbiological and clinical diagnosis rates observed in this study was large during the validation period, and similar findings have also been reported from other settings [18]. Second, the telenursing data were based on chief complaint codes defined for Sweden. Some complaints, such as fever and cough, were coded as age-specific syndromes, while other complaints had an age-neutral coding. Internationally standardized telenursing complaint codes would facilitate valid and reliable recording and comparisons between telenursing systems. Third, while data from telenursing centers and website usage data from health service providers were prospectively collected, the GFT data were downloaded in 2012. It is not known if the GFT data for 2007-2011 had been retrospectively adjusted to better correlate to recorded ILI rates. Although it has been reported that GFT algorithms are recalibrated every year [6,23], it is not evident whether or not these recalibrations influence the transformed Web query data available for download. Fourth, this study analyzed the correlations between trends in a set of eHealth data sources and influenza case data. For use in surveillance practice, algorithms need to be developed to translate the time series data into actionable alerts [27]. Finally, it should not be forgotten that different strains of the influenza virus affect different age groups and that eHealth surveillance may be less reliable during winter influenza seasons when the circulating influenza strains mainly affects the elderly in the population.

### Conclusions

We found correlations with large effect sizes between eHealth data and influenza case rates in a representative Swedish county over a five-year period including both winter influenza seasons and a pandemic influenza outbreak. Both telenursing center and page-specific website usage data performed at the level of GFT. Although the study design does not allow us to draw conclusions about causal associations with media coverage, we observed that a two-week time lag between eHealth data sources and influenza rates was reduced to one week or none from the 2009 pandemic outbreak when there was parallel intense media coverage. Similarly, we found a tendency for eHealth surveillance to perform worse during winter influenza seasons when the influenza activity involved adolescents and young adults to a lesser degree. The main theoretical implications of the study are that analytic methods need to be developed that adjust eHealth surveillance system to shifts in media coverage and variations in age-group related immunity to specific virus strains. The practical inference is that further longitudinal research incorporating prospective evaluations of actionable alerts [28] is required before eHealth surveillance systems can be used in routine public health practice.

## Abbreviations

A: influenza A
EHR: electronic health record
GA: Google Analytics
GFT: Google Flu Trends
ILI: influenza-like illness
RIR: relative illness ratio
